# Development and evaluation of an educational comic leaflet for pediatric anesthesia care

**DOI:** 10.1016/j.bjane.2024.844521

**Published:** 2024-05-29

**Authors:** Fabiano Soares Carneiro, Juliana Lacerda de Oliveira Campos, Stephanie Bruna Camilo Soares de Brito, Flávia Marques de Melo, Eliane Cristina de Souza Soares

**Affiliations:** a TSA/SBA, Grupo SAM (Serviço de Anestesiologia e Medicina) – Rede Mater Dei de Saúde, Belo Horizonte, MG, Brazil; b Universidade Federal de Minas Gerais, Belo Horizonte, MG, Brazil; c Grupo SAM (Serviço de Anestesiologia e Medicina) – Rede Mater Dei de Saúde, Belo Horizonte, MG, Brazil

*Dear Editor,*

Preoperative anxiety has been reported in over 60% of children undergoing anesthesia.^1^^,^^2^ High levels of preoperative anxiety reduce the ability to cooperate during anesthesia and appear to be associated with an increased incidence of emergence delirium,^1^ decreased pain threshold leading to increased postoperative pain, and development of postoperative mal-adaptive behavioral changes such as enuresis, apathy, social isolation, sleep and eating disorders.^2^ Preoperative anxiety has therefore been addressed in various ways, including pharmacological and non-pharmacological methods.

The use of preanesthetic medications such as midazolam, ketamine, and dexmedetomidine are possible pharmacological strategies and should always be considered and discussed but have specific indications and are not exempt of adverse events. In turn, play therapy, comic strips, and video games are examples of non-pharmacological approaches that may have relatively low negative outcomes.^3^^,^^4^ The literature regarding this set of approaches is limited, but some interesting results have been shown worldwide. For instance, a cartoon-based anesthesia information leaflet has effectively reduced preoperative anxiety in children aged 6 to 17 years who were candidates for elective surgery and was considered an interesting and cost-effective tool.^2^

Resolution 1.274/2017 of the Brazilian Federal Council of Medicine, regarding the practice of anesthesia, proposes that all patients, including children, must undergo a preoperative anesthesia visit before being submitted to elective procedures. This is an opportunity for the anesthesiologist to introduce the leaflet and interact with children in a way that can have a positive impact on reducing anxiety and its poor outcomes on the day of surgery.

A literature review over leaflets and preoperative care was carried out in the MEDLINE database. This was fundamental for selecting the leaflet's content, as well as determining the formatting used to present it to the pediatric audience in the preoperative setting. A team member wrote the text using the information collected, anesthesia team routines, as well as input from childcare and education experts. This text was presented to children in the targeted age group and some adaptations were adopted based on their insights. To stimulate interest and increase interaction with the contents of the leaflet, a mascot was designed, and pictures were drawn to help introduce the information. A specific space where children could actively participate in the story and draw their desired dream while sleeping under anesthesia was included (Fig. 1). After elaboration, the leaflet was evaluated according to the BALD criteria (Baker Able Leaflet Design) by the research team.^5^ The leaflet created achieved 27 points, which is considered sufficient in terms of layout and design.

**Figure 1:**
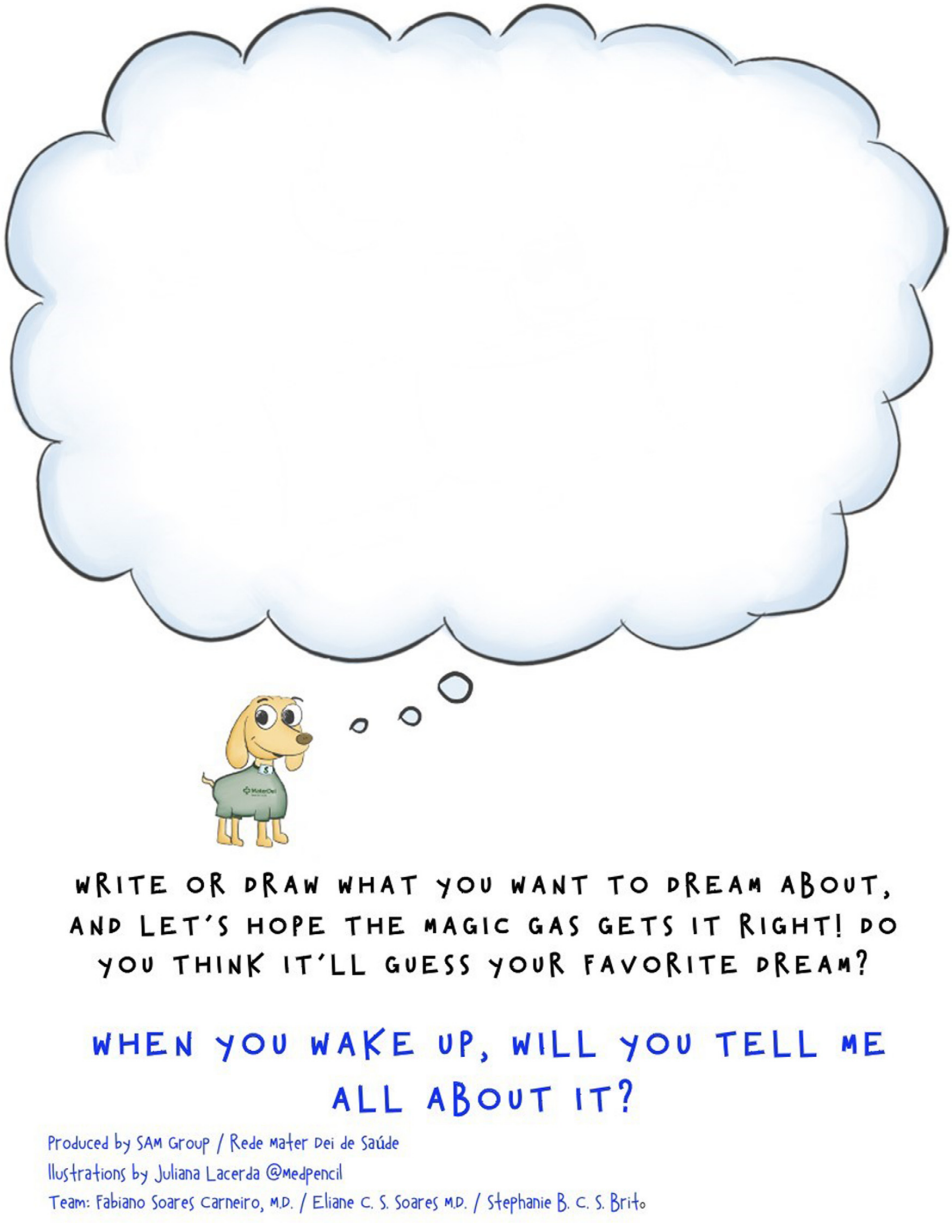
Space in the leaflet for children to participate in the story.

Approval was obtained from the anesthesia team, as well as pediatric surgeons and pediatric specialists. Subsequently, 87 leaflets were printed and distributed to children during the preoperative anesthesia examination that occurred from one month to at least one day prior to surgery. The patients were between 2 and 8 years old and had no diseases or conditions that led to delays in neuropsychomotor development. All selected children were accompanied by a caregiver who was able to read and understand the contents of the brochure. The caregiver was informed about the study. Within 24 hours of delivery of the brochure, a telephone text message containing a questionnaire was sent by a research assistant to the caregiver who attended the consultation. The questionnaire contained 4 questions, and the average response time was about 3 minutes. In case the questionnaire was not answered within 48 hours, a researcher actively reached out to the family and asked for their engagement.

A total of 52.7% of caregivers answered the questionnaire. The mean age of the addressed children was 4.4 years old, and the most prevalent surgery type was adenoidectomy (64.4%). The introduction of the anesthetic act in an appointment prior to surgery day using a comic strip leaflet was considered useful by 99.98% of participants. The leaflet itself was positively received by caregivers as 91.5% of them declared they enjoyed it. There were no significant concerns declared by caregivers. Suggestions given included the development of a video with the leaflet's content and the comic strip's character physically present on the day of surgery.

Our results show that information prior to surgery regarding the anesthetic act may be well accepted by children and caregivers when presented as a comic strip leaflet. Thus, we concluded that the leaflet was found to be accessible, informative and useful. Although the reduction in anxiety level was not addressed in the present study, the positive response to this initiative can lead to further investigation regarding this matter.

## Conflicts of interest

The authors declare no conflicts of interest.
